# Surgical outcomes between posterior decompression alone and posterior decompression with fusion surgery among patients with Meyerding grade 2 degenerative spondylolisthesis: a multicenter cohort study

**DOI:** 10.1186/s12891-022-05850-4

**Published:** 2022-10-08

**Authors:** Keiichiro Tozawa, Yoshitaka Matsubayashi, So Kato, Toru Doi, Yuki Taniguchi, Yudai Kumanomido, Akiro Higashikawa, Yuichi Yosihida, Naohiro Kawamura, Katsuyuki Sasaki, Seiichi Azuma, Jim Yu, Nobuhiro Hara, Masaaki Iizuka, Takashi Ono, Masayoshi Fukushima, Yujiro Takeshita, Sakae Tanaka, Yasushi Oshima

**Affiliations:** 1 Department of Orthopaedic Surgery, Faculty of Medicine, The University of Tokyo, 7-3-1, Hongo, Bunkyo-Ku, 113-8655 Tokyo, Japan; 2 Department of Orthopaedic Surgery, Japan Organization of Occupational Health and Safety Kanto Rosai Hospital, 1-1, Kizukisumiyoshi-Cho, Nakahaha-Ku, 211-8510 Kawasaki City, Kanagawa Japan; 3 Department of Spine and Orthopedic Surgery, Japanese Red Cross Medical Center, 4-2, Hiroo, Shibuya- Ku, 150-8935 Tokyo, Japan; 4 Department of Orthopaedic Surgery, Japanese Red Cross Saitama Hospital, 1-5, Shintoshin, Chuo-Ku, 330-8553 Saitama City, Saitama Japan; 5 Department of Orthopaedic Surgery, Japanese Red Cross Musashino Hospital, 1-26-1, 180-0023 Kyonancho, Musashino City, Tokyo Japan; 6 Department of Spinal Surgery, Japan Community Health-care Organization Tokyo Shinjuku Medical Center, 5-1, Tsukudo-Cho, Shinjuku-Ku, 162-8543 Tokyo, Japan; 7 Spine Center, Toranomon Hospital, 2-2-2, Toranomon, Minato-Ku, 105-8470 Tokyo, Japan; 8 Department of Orthopaedic Surgery, Japan Organization of Occupational Health and Safety Yokohama Rosai Hospital, 3211, Kozukue-Cho, Kohoku-Ku, 222-0036 Yokohama City, Kanagawa Japan

**Keywords:** Meyerding grade 2 degenerative spondylolisthesis, Multicenter retrospective cohort study, Central canal stenosis, Decompression, Fusion, Patient-reported outcome measure

## Abstract

**Background:**

Whether lumbar decompression with fusion surgery is effective against Meyerding grade 2 degenerative spondylolisthesis (DS) is unknown. Therefore, the current study aimed to compare the surgical outcomes between posterior decompression alone and posterior decompression with fusion surgery among patients with grade 2 DS with central canal stenosis.

**Methods:**

This retrospective cohort study included prospectively registered patients (n = 3863) who underwent surgery for degenerative lumbar spinal canal stenosis at nine high-volume spine centers from April 2017 to July 2019. Patients with grade 2 DS and central canal stenosis were included in the analysis. Patients with radiculopathy, including foraminal stenosis, degenerative scoliosis, and concomitant anterior spinal fusion, and those with a previous history of lumbar surgery were excluded. The participants were divided into the decompression alone group (group D) and decompression with fusion surgery group (group F). Data about patient-reported outcomes, including Numeric Rating Scale (low back pain, leg pain, leg numbness, and foot numbness), Oswestry Disability Index, EuroQol Five-Dimensional questionnaire, and 12-Item Short-Form Health Survey scores, were obtained preoperatively and 2 years postoperatively.

**Results:**

In total, 2354 (61%) patients, including 42 (1.8%) with grade 2 DS (n = 18 in group D and n = 24 in group F), completed the 2-year follow-up. Group D had a higher proportion of female patients than group F. However, the two groups did not significantly differ in terms of other baseline demographic characteristics. Group D had a significantly shorter surgical time and lower volume of intraoperative blood loss than group F. Postoperative patient-reported outcomes did not significantly differ between the two groups, although the preoperative degree of low back pain was higher in group F than in group D. The slip degree of group D did not worsen during the follow-up period.

**Conclusion:**

The surgical outcomes were similar regardless of the addition of fusion surgery among patients with grade 2 DS. Decompression alone was superior to decompression with fusion surgery as it was associated with a lower volume of intraoperative blood loss and shorter surgical time.

## Background

Lumbar decompression with or without fusion surgery is one of the most common surgical interventions for treating degenerative lumbar spinal canal stenosis (DLSS). Degenerative spondylolisthesis (DS) is as a condition in which one vertebra slips over the other due to facet arthropathy and intervertebral disc degeneration [1]. This condition is often observed in patients with DLSS [2].

A large randomized control trial (RCT) showed that surgical management, compared with conservative treatment, improves pain and function among patients with symptomatic DS [3]. According to a previous report, 96% of patients with DS undergo fusion surgery as an adjunct to decompression surgery in the United States [4]. Another RCT directly compared laminectomy plus fusion versus laminectomy alone in patients with grade 1 DS. Results showed that compared with laminectomy alone, the addition of lumbar spinal fusion to laminectomy was associated with better surgical outcomes [5]. However, there are still controversies in terms of the appropriate surgical procedure (whether to perform fusion of the affected vertebrae) for DS with symptomatic stenosis. Two RCTs reported that decompression alone was not inferior to decompression with fusion surgery among patients with low-grade (Meyerding grade 1 or 1–2) DS [6, 7].

Previous reports exhibited heterogeneity in terms of slippage grade. Surgical outcomes could differ between patients with grade 1 and 2 DS, and it was hypothesized that fusion surgery is better for patients with grade 2 DS. However, no study focused on patients with grade 2 DS. In addition, patients with foraminal stenosis should be excluded because they generally require facetectomy with posterior fusion surgery or lateral interbody fusion surgery [8]. Therefore, the current study aimed to compare surgical outcomes between decompression alone and decompression with fusion surgery among patients with DLSS accompanied by grade 2 DS. To achieve a homogenous population, this research focused on patients with grade 2 DS who presented with central canal stenosis without radiculopathy.

## Methods

### Patients

This multicenter retrospective cohort study included prospectively registered patients who underwent surgery for DLSS at nine high-volume spine centers from April 2017 to July 2019. The inclusion criteria were as follows: (1) patients who underwent posterior lumbar surgery for single-level DLSS with grade 2 DS and (2) those with central canal stenosis with neurologic symptoms such as weakness and numbness in the bilateral lower extremities, intermittent claudication, and bladder and bowel dysfunction. The exclusion criteria were as follows: (1) patients with unilateral radiculopathy including foraminal stenosis, (2) those with degenerative scoliosis (Cobb angle of ≥ 10°), (3) those with concomitant anterior spinal fusion (including lateral interbody fusion), and (4) those with a previous history of lumbar surgery.

The patients were divided into the decompression alone group (group D) and decompression with fusion surgery group (group F). Surgical procedures were selected independently by each surgeon. The study protocol was assessed and approved by the institutional review boards of each hospital. All participants provided a written informed consent.

### Background characteristics and Surgical Data

Clinical data such as age, sex, body mass index (BMI), history of diabetes mellitus (DM), rheumatoid arthritis, and smoking were collected from our database. Surgical factors included operative time, volume of intraoperative blood loss, and any adverse event during surgery such as an unplanned dural tear and nerve damage. Information about postoperative hospitalization and complications, including surgical site infection, symptomatic hematoma at the surgical site, respiratory tract infection, urinary tract infection, cardiovascular and cerebrovascular disorders, and death, occurring within 30 days after surgeries was also recorded.

### Radiologic evaluation

All patients underwent radiographic examinations, with an anteroposterior view and three lateral views (maximally flexed position, neutral position, and maximally extended position) both preoperatively and 2 years postoperatively. The presence of DS was assessed via neutral lateral radiography and calculation of slip degree (mm) and percentage of slip (%). DS was defined as the presence of a vertebra that slipped forward in relation to the vertebra below it. Then, the degree of slip rate was categorized according to the Meyerding classification, with grades I, II, III, and IV indicating 25%, 50%, 75%, and 100% displacement, respectively [9].

Intervertebral disc height (mm) was assessed via standing neutral lateral radiography and was defined as the average of the anterior and posterior margins of the intervertebral space. Moreover, the difference in slip degree between the flexion and extension positions was calculated on lateral radiography as Δslip (mm). The slip degree was evaluated 2 years after surgery in both groups, and the fusion rate of group F was assessed. A successful fusion was defined using the criteria of both dynamic radiography and CT scan, including translation of ≤ 3 mm and angular motion of ≤ 5° during flexion/extension on lateral lumbar radiography and the presence of bridging trabecular bone within the disk space in the absence of loosening of the pedicle screws on CT scan [10, 11].

### Clinical outcomes

Data about patient-reported outcomes (PROs), including Numeric Rating Scale (NRS) (low back pain, leg pain, leg numbness, and foot numbness), Oswestry Disability Index (ODI), EuroQol Five-Dimensional (EQ-5D) questionnaire, and 12-Item Short-Form Health Survey (SF-12), were obtained preoperatively and 2 years postoperatively.

### Statistical analysis

The Wilcoxon rank-sum test and Pearson’s χ2 test were used to analyze continuous and categorical variables, respectively. All statistical analyses were performed using JMP software version 15.1.0 (SAS Institute Inc., Cary, North Carolina, the USA). P-values of < 0.05 were considered statistically significant.

## Results

### Patients

**Fig. 1:**
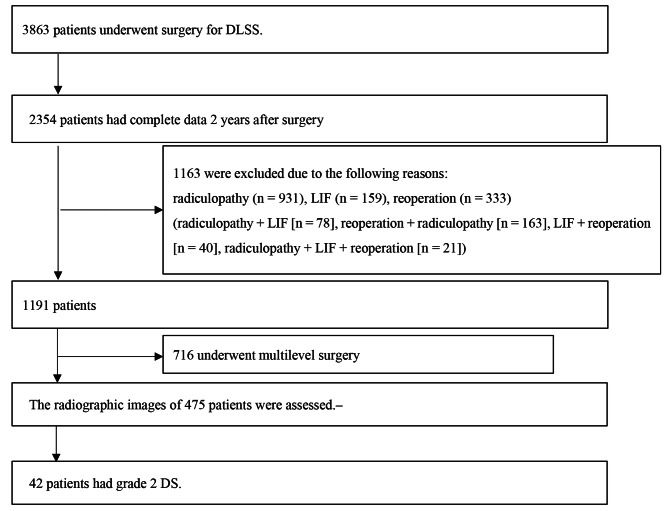
Selection of participants In total, 3863 consecutive patients underwent surgery for DLSS. The clinical outcomes of 2354 (61%) patients 2 years after surgery were assessed. Among them, 1163 who did not meet our criteria and 716 who had multilevel surgery were excluded. Therefore, the radiographic images of the remaining 475 patients were evaluated, and 42 patients were found to have grade 2 DS

In total, 3863 consecutive patients underwent surgery for DLSS (Fig. 1). Among them, 2354 (61%) patients completed the 2-year follow-up. Among them, 475 underwent single-level posterior decompression surgery with or without fusion. Based on preoperative radiography, 42 patients were found to have grade 2 DS and were divided into the decompression alone group (group D, n = 18) and decompression with fusion surgery (group F, n = 24). The mean age at surgery was 70.3 (range: 46–84) years, and the mean BMI was 23.0 (range: 18.1–30.9) kg/m^2^. In total, nine (21%) patients were men. The mean surgical time was 145 (range: 32–279) min; mean volume of intraoperative blood loss, 180 (range: 0–630) mL; and mean length of postoperative hospitalization, 13.1 (range: 5–53) days.

Table 1 shows the demographic characteristics of each group. Group D had a higher proportion of female patients than group F (*P* < 0.01). However, the other baseline demographic characteristics did not significantly differ between the two groups (*P* > 0.05). Group D had a significantly shorter surgical time and lower volume of intraoperative blood loss than group F (*P* < 0.01). Moreover, the length of postoperative hospitalization was significantly shorter in group D than in group F (*P* < 0.01).

**Table 1:** Demographic characteristics and surgical factors between groups D and F

	Group D (n = 18)	Group F (n = 24)	P value
Age	70.4 ± 8.7	70.2 ± 9.1	0.88
Sex (male:female)	8 : 10	1 : 23	< 0.01
BMI (kg/m^2^)	23.4 ± 3.8	22.8 ± 2.7	0.97
ASA (≤ 2:3)	18:0	23:1	0.38
DM (n)	0 (0%)	4 (17%)	0.07
RA (n)	1 (6%)	1 (4%)	0.83
Smoking (n)	1 (6%)	2 (8%)	0.73
Surgical duration (min)	87.4 ± 40.2	188.2 ± 48.0	< 0.01
Volume of intraoperative blood loss (mL)	73.6 ± 105.5	259.2 ± 163.3	< 0.01
Perioperative complication dural tear (n) surgical site infection (n)	1 (6%) 0 (0%)	0 (0%) 1 (4%)	0.24 0.38
Length of postoperative hospitalization (days)	8.1 ± 3.0	16.8 ± 8.9	< 0.01

Values were expressed as means ± SD. The Wilcoxon rank-sum test and the Pearson’sχ2 test were used to assess continuous and categorical variables, respectively. A P value of < 0.05 was considered statistically significant

BMI, Body mass index; ASA, American Society of Anesthesiologists; DM, diabetes mellitus; RA, rheumatoid arthritis

### Radiologic results

Table 2 depicts the radiologic results. There was no significant difference between groups D and F in terms of preoperative slip degree (*P* = 0.69), percentage of slip (*P* = 0.27), preoperative disk height (*P* = 0.42), and preoperative dynamic Δslip (*P* = 0.72). The slip degree of group D did not worsen during the follow-up period (*P* = 0.35). Meanwhile, the slippage significantly improved postoperatively (*P* < 0.01), with a fusion rate of 83.3% in group F.

**Table 2:** Radiographic outcomes between groups D and F

	Group D (n = 18)	Group F (n = 24)	P value
Operated segments			0.40
L3–L4 (n)	1	0	
L4–L5 (n)	16	21	
L5–S1 (n)	1	3	
Preoperative disc height (mm)	5.3 ± 1.6	4.8 ± 1.8	0.42
Preoperative dynamic Δslip (mm)	2.1 ± 2.0	1.7 ± 1.3	0.72
Preoperative percentage of slip (%)	26.2 ± 1.1	27.0 ± 2.0	0.27
Preoperative amount of slippage (mm)	10.0 ± 0.8	10.3 ± 1.2	0.69
Postoperative amount of slippage (mm)	10.4 ± 0.9	6.2 ± 2.7	< 0.01
Fusion rate (%)	-	83.3 (20/24)	-

Values were expressed as means ± standard deviation

The Wilcoxon rank-sum test and the Pearson’sχ2 test were used to assess continuous and categorical variables, respectively

A P value of < 0.05 was considered statistically significant

### Complications

In terms of perioperative complications, one patient in group D presented with dural tear and one in group F with surgical site infection. None of the patients in both groups had symptomatic hematoma, respiratory tract infection, urinary tract infection, and cardiovascular or cerebrovascular disorders. Moreover, there was no 30-day mortality after the surgeries, and no patient required additional surgery during the follow-up period.

### Clinical outcomes

Table 3 shows the clinical outcomes of groups D and F. Group F had a significantly higher preoperative NRS score for low back pain than group D (*P* = 0.04). However, the results did not significantly differ postoperatively. None of the other evaluation scores differed between the two groups.

**Table 3:** Clinical outcomes between groups D and F

Outcome		Group D (n = 18)	Group F (n = 24)	P value
NRS low back pain	Preoperative	3.8 ± 2.5	5.9 ± 3.2	0.04
	Postoperative	2.0 ± 2.3	2.8 ± 2.8	0.40
	Extent of change	1.8 ± 1.6	3.1 ± 4.4	0.31
NRS leg pain	Preoperative	6.0 ± 3.6	5.3 ± 4.1	0.82
	Postoperative	2.6 ± 1.9	1.7 ± 2.0	0.13
	Extent of change	3.4 ± 3.6	3.6 ± 4.5	0.98
NRS leg numbness	Preoperative	3.8 ± 3.1	4.8 ± 3.5	0.35
	Postoperative	2.6 ± 2.1	1.8 ± 2.6	0.12
	Extent of change	1.2 ± 4.3	3.0 ± 4.2	0.20
NRS foot numbness	Preoperative	3.1 ± 2.8	4.3 ± 3.3	0.26
	Postoperative	2.3 ± 2.4	2.8 ± 3.5	0.61
	Extent of change	0.8 ± 2.4	1.5 ± 3.0	0.48
SF-12 PCS	Preoperative	25.4 ± 15.5	26.2 ± 13.8	0.77
	Postoperative	39.6 ± 18.0	39.2 ± 14.1	0.73
	Extent of change	14.2 ± 21.2	13.0 ± 18.6	0.94
EQ-5D	Preoperative	0.55 ± 0.13	0.57 ± 0.17	0.70
	Postoperative	0.74 ± 0.15	0.75 ± 0.18	0.90
	Extent of change	0.18 ± 0.17	0.20 ± 0.24	0.88
ODI	Preoperative	39.5 ± 17.7	42.2 ± 16.0	0.31
	Postoperative	14.4 ± 14.2	17.5 ± 17.4	0.55
	Extent of change	25.1 ± 22.5	24.8 ± 17.3	0.76

Values were expressed as means ± SD. The Wilcoxon rank-sum test was used to assess continuous variables. A P value of < 0.05 was considered statistically significant

NRS, Numerical Rating Scale; SF-12, Short Form-12; PCS, Physical Component Summary; EQ-5D, EuroQol 5 Dimension; ODI, Oswestry Disability Index

## Discussion

The current study aimed to compare the surgical outcomes between decompression alone surgery and decompression with fusion surgery among patients with grade 2 DS. Consequently, the clinical outcomes were similar between the two group D and F 2 years after surgery. However, decompression alone was associated with less invasiveness in terms of surgical time and volume of intraoperative blood loss. To the best of our knowledge, this was the first study that compared the efficacy of fusion surgery and decompression alone among patients with grade 2 spondylolisthesis.

Moreover, the PROs of decompression alone were similar to those of decompression with fusion surgery 2 years after surgery. However, group D had a shorter surgical time and volume of intraoperative blood loss than group F. This finding was in accordance with that of a previous RCT conducted by Austevoll et al., which compared decompression alone and decompression with fusion surgery among patients with lumbar spinal stenosis and DS [7]. They randomly included 267 patients with DS, and their 2-year follow-up was completed with 216 patients. The PROs were similar between the two groups both preoperatively and postoperatively, and the reoperation rate did not differ. Meanwhile, decompression alone is associated with a shorter surgical time and shorter length of hospital stay. In addition, several reports revealed higher risks of severe complications after fusion surgery. A large analysis of registry data revealed that the addition of fusion surgery to decompression surgery doubled the risk of severe adverse events [12]. Moreover, patients aged over 80 years can have a higher incidence of complications than younger patients [13]. Rosen et al. revealed that less-invasive lumbar decompression was not correlated with major complications among elderly patients [14]. Therefore, since decompression alone is less invasive and is associated with a shorter duration of hospitalization, it can be a better option particularly among elderly patients with grade 2 DS.

In the current study, patients who did not undergo fusion surgery did not present with slippage deterioration postoperatively. There was no worsening of slippage after decompression for grade 1 DS [15–17]. By contrast, Matsunaga et al. showed that 34% of patients with DS experienced slippage progression within the natural course of disease (> 10 years) [18]. In the current study, the degenerated disc might be the end stage of degeneration as evidenced by the low disc height, which we speculate made the intervertebral slippage stable. However, with consideration of the 2-year postoperative follow-up period in this study, surgeons must keep in mind that slippage may worsen in the long-term after decompression. Moreover, patients who underwent decompression and fusion surgery may further require additional surgeries including instrument removal and operation at the adjacent segment level. Nevertheless, surgeons should cautiously consider long-term follow-up.

Patients who underwent fusion surgery were more likely to have a higher degree of preoperative low back pain. This may represent the preference of surgeons in selecting fusion surgery among patients with low back pain. That is, surgeons were more likely to perform fusion surgery on patients with DS due to the belief that low back pain could be caused by slippage itself and could be treated via fusion surgery. Indeed, Sengupta et al. reported that low back pain may be attributed to intervertebral disc or facet joint degeneration. Hence, fusion is required in such cases [19]. Moreover, Kleinstueck et al. showed that low back pain improved with decompression with fusion surgery among patients with DS [20]. However, Matsudaira et al. revealed that low back pain can improve with decompression alone [21]. Austevoll performed an RCT, and results showed that the degree of low back pain improvement was similar regardless of the presence of fusion surgery [7]. The postoperative degree of low back pain did not differ between the two groups in this study. Therefore, further studies must be conducted to identify whether fusion surgery is important in terms of reducing low back pain in patients with grade 2 DS.

The current study had several limitations. First, the indications for surgical procedures were not randomized. In addition, owing to the retrospective nature of the study design, the detailed surgical procedure depended on the preference by each surgeon. Second, the sample size in this study was small (n = 42). To achieve a heterogenous population, we only selected patients with central canal stenosis to exclude the risk of foraminal stenosis, which commonly requires fusion surgery. Therefore, our results may not be representative of most patients with lumbar spinal stenosis treated in actual clinical practice. Third, the follow-up period was only 2 years, which may be extremely short for calculating the rates of additional surgeries. Fourth, the postoperative follow-up rate was relatively low. Further, we only included patients with data about radiographic examinations and PROs 2 years after surgery, and this led to a decrease in the number of participants. Thus, further prospective studies should be conducted to address these issues. Nevertheless, since only 42 (1.8%) of 2354 patients met the inclusion criteria, we believe this research is strong in that we could obtain homogenous population with grade 2 spondylolisthesis, which is a relatively rare condition.

## Conclusion

There were no significant differences in terms of surgical outcomes between decompression alone and decompression with fusion surgery 2 years after surgery among patients with grade 2 DS with central canal stenosis. Decompression alone was considered less invasive and was associated with a lower volume of intraoperative blood loss and shorter surgical time.

## Data Availability

The datasets generated and/or analyzed during the current study are not publicly available due to their containing information that could compromise the privacy of research participants but are available from the corresponding author on reasonable request.

## Abbreviations

DLSS: degenerative lumbar spinal canal stenosis
DS: degenerative spondylolisthesis
RCT: randomized control trial
BMI: body mass index
DM: diabetes mellitus
PROs: patient-reported outcomes
NRS: Numeric Rating Scale
ODI: Oswestry Disability Index
EQ-5D: EuroQol Five-Dimensional
SF-12: Short-Form Health Survey
